# Increased *Borrelia burgdorferi* Seroprevalence in Nova Scotia—Prevalence and Distribution 10 Years Later

**DOI:** 10.1111/zph.70033

**Published:** 2025-12-02

**Authors:** Carrie Phillips, Colleen Jackson, Linda Passerini, Kathryn McIsaac, Courtney Loomer, Heather Coatsworth, Jennifer Cram, Elizabeth Simms, David Haldane, Todd F. Hatchette, Glenn Patriquin

**Affiliations:** ^1^ National Microbiology Laboratory Public Health Agency of Canada Winnipeg Manitoba Canada; ^2^ Provincial Public Health Laboratory Network Nova Scotia Canada; ^3^ Division of Microbiology, Department of Pathology and Laboratory Medicine Nova Scotia Health Halifax Nova Scotia Canada; ^4^ Department of Health and Wellness Public Health Halifax Nova Scotia Canada; ^5^ National Microbiology Laboratory, Mycobacteriology, Vector‐Borne, and Prions Diseases Division Winnipeg Manitoba Canada; ^6^ Department of Pathology Dalhousie University Halifax Nova Scotia Canada

**Keywords:** *Borrelia burgdorferi*, Lyme disease, Nova Scotia, serosurvey, serum

## Abstract

Lyme disease (LD), a tick‐borne infection, is endemic in Nova Scotia. One decade ago, the seropositivity rate to *Borrelia burgdorferi* was 2/1855 (0.14%). In the current study, using residual sera representing ages 10–64 years, we demonstrate an increase in seroprevalence, to 25/1872 (1.60%), with the highest seroprevalence in the western area of the province.

## Impacts

- Seroprevalence of *Borrelia burgdorferi* in Nova Scotia has increased as compared to 10 years ago
- Seropositivity is detected throughout the province
- Highest rates of seropositivity are in the province's Western Zone

## 1 Introduction

The incidence of Lyme disease (LD), caused by *Borrelia* (*Borreliella*) *burgdorferi* sensu stricto, is increasing in Canada, and it is a reportable disease in Nova Scotia, a province with the highest incidence of LD in the country (Public Health Agency of Canada 2023). In Eastern Canada and the Northeastern United States, *B. burgdorferi* is transmitted by the black‐legged deer tick ( *Ixodes scapularis* ). In 2012, a serosurvey was performed, using residual sera in Nova Scotia (specimens from routine outpatient testing that would have otherwise been discarded). Anonymised samples representing males and females, aged 10–64 years, from each District Health Authority were tested using the standard two‐tiered testing (STTT) algorithm and revealed a seropositivity rate of 2/1855 (0.14%) (Hatchette et al. 2015). The entire province of Nova Scotia has since become an at‐risk area for LD and the number of annual clinical cases reported to public health has increased from 2 cases in 2002 (Nova Scotia Department of Health 2003) to over 2000 cases in 2023 (Nova Scotia Department of Health and Wellness 2024). Although the significant increase is partially related to the change in the case definition used by Public Health (relying solely on laboratory data rather than clinical evidence coupled with laboratory confirmation), given this continued increase in cases, we sought to determine the seroprevalence of exposure to *B. burgdorferi* in Nova Scotia, as an update to prior work.

## 2 Materials and Methods

After Institutional Research Ethics Board approval, we collected 1852 residual serum samples that would have otherwise been discarded after diagnostic testing, similar to the prior study (Hatchette et al. 2015). Nine regional laboratories from throughout the province provided deidentified residual sera originally submitted for routine testing such as prenatal bloodwork, HIV, cholesterol and endocrinology requests between October 2022 and November 2023. Specimens were stratified by patient age, sex and former District Health Authority (fDHA) to maintain consistency with the 2012 data. The fDHAs have since been amalgamated into a single provincial health authority, divided into administrative zones (Western [fDHAs 1–3], Northern [fDHAs 4–6], Eastern [fDHAs 6–8] and Central [fDHA 9]). To best replicate the previous study (Hatchette et al. 2015), we used sample sizes determined previously (based on estimated seroprevalence of 1.0% ± 0.5% precision), representing the population proportionate to its fDHA (with oversampling in the Western Zone) and ensuring representation of sexes and age groups (10‐year age groups from 10 to 59 years and a single age group from 60 to 64 years).

The modified two‐tiered testing (MTTT) algorithm was performed as described previously (Khan et al. 2022; Davis et al. 2020). Briefly, a commercially available ZEUS pepC10/VlsE enzyme immunoassay (EIA) (ZEUS ELISA *Borrelia* VlsE1/pepC10 IgG/IgM, ZEUS Scientific, Branchburg, NJ) was used for screening the sera. Specimens that were reactive or equivocal to the screen were then confirmed as positive with the Zeus whole cell sonicate EIA (ZEUS ELISA *Borrelia burgdorferi* IgG/IgM). This reactive cohort was also tested approximating the STTT method (noting that the previous STTT method used the WCS EIA as an initial screen). Briefly, the pepC10/VlsE EIA‐reactive samples were tested with a *B. burgdorferi* US IgG immunoblot (EUROIMMUN Medical Diagnostics Canada Inc., Mississauga, ON) at the National Microbiology Laboratory (Winnipeg, Canada), according to the manufacturer's instructions. A positive IgG immunoblot consisting of at least 5/10 significant bands (p18/21, p25 (OspC), p28, p30, p39 (BmpA), p41, p45, p58, p66 or p83/93) was considered reactive, while a borderline (BL) result was defined as 4/10 significant positive bands, in addition to at least one weakly positive significant band or, when 4/10 significant bands plus the VlsE band were positive. Five BL results were considered positive for the analysis to match previously used methods.

Similar to the prior serosurvey, descriptive statistics were used as previously (Hatchette et al. 2015), with proportions calculated with 95% CI by the Clopper–Pearson Exact method, using EPITOOLS (https://epitools.ausvet.com.au) and GraphPad QuickCalcs (https://www.graphpad.com/quickcalcs), with oversampling in the Western Zone to best approximate the previous work.

## 3 Results

**FIGURE 1:**
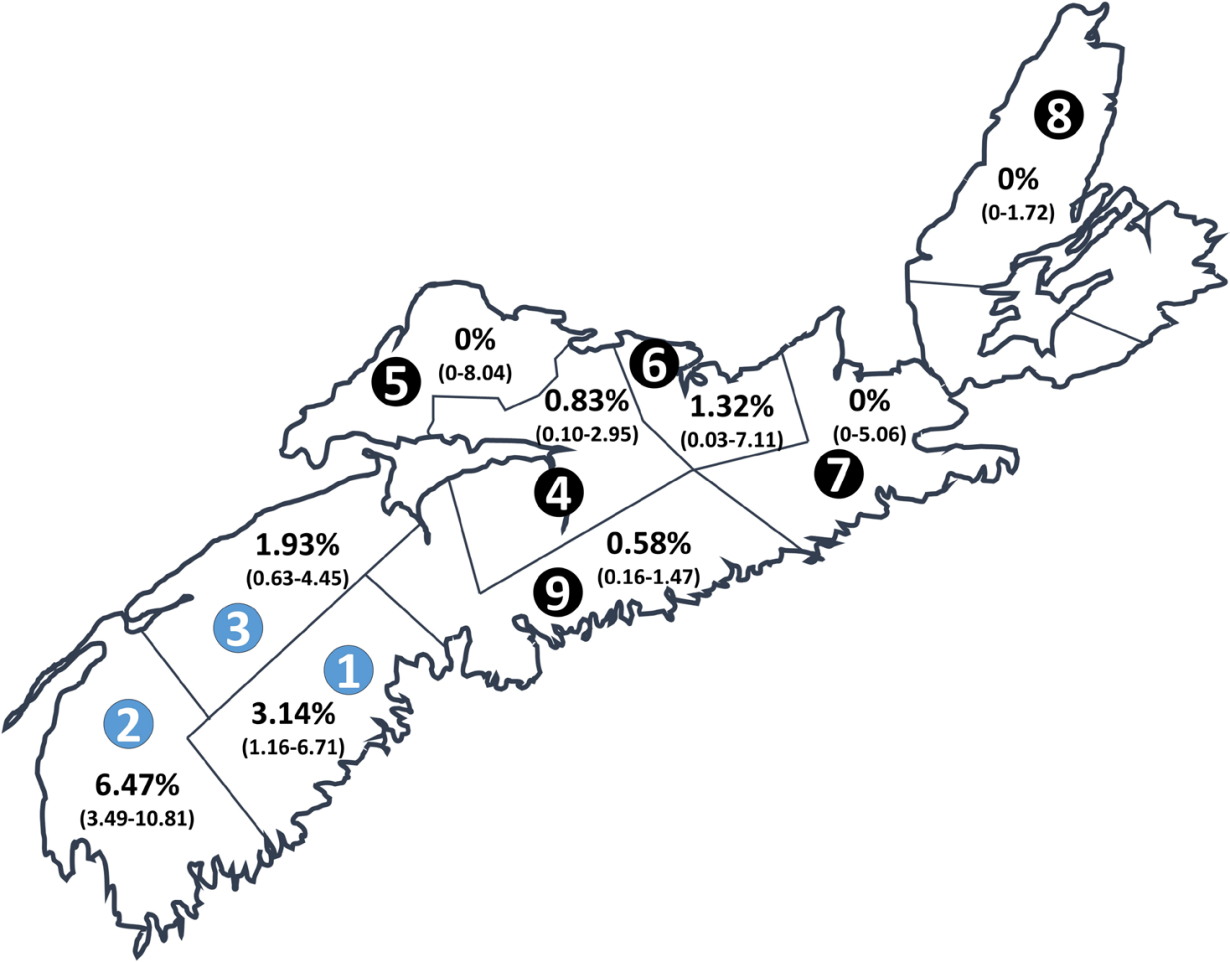
Map of Lyme seropositivity in Nova Scotia, Canada. Former District Health Authorities (fDHAs) are numbered in circles. Seroprevalence for each fDHA is expressed as a percentage, with corresponding 95% confidence interval in parentheses. fDHAs with blue numbered circles represent the province's Western Zone.

Employing the MTTT assay, we screened 1872 serum specimens. A total of 93 specimens were positive or indeterminate by the pepC10/VlsE EIA and underwent confirmation testing by the WCS EIA (Table 1). Of the 93 screen‐positive samples, 30 were confirmed with the WCS EIA (MTTT assay), for a total seroprevalence of *B. burgdorferi* in Nova Scotia ranging from 0% to 6.47%, depending on the fDHA (Figure 1). Province‐wide, seroprevalence was found to be 1.60% (95% CI 1.08–2.28) overall. The highest seroprevalence was found in the Western Zone (fDHAs 1–3), at 3.68% (95% CI 2.37–5.42), especially fDHA 2, at 6.47% (95% CI 3.49–10.81). Among the 30 positive patients, two‐thirds were male, and there was no significant trend by age.

**TABLE 1:** Seroprevalence of *B. burgdorferi* in Nova Scotia, by assay and region.

Region	Total screening tests	pepC10/VlsE EIA positive or indeterminate, no.	WCS EIA positive or indeterminate, no.	Estimated seroprevalence based on MTTT (95% CI)	IgG immunoblot positive or borderline, no.	Estimated seroprevalence based on IgG immunoblot (95% CI)
Western Zone	653	46	24	3.68 (2.37–5.42)	21	3.22 (2.0–4.87)
fDHA 1	191	16	6	3.14 (1.16–6.71)	5	2.62 (0.86–6.0)
fDHA 2	201	18	13	6.47 (3.49–10.81)	11	5.47 (2.76–9.58)
fDHA 3	259	12	5	1.93 (0.63–4.45)	5	1.93 (0.63–4.45)
Northern Zone	242	12	2	0.83 (0.10–2.95)	1	0.41 (0.01–2.28)
fDHA 4	122	7	1	0.82 (0.02–5.93)	0	0 (0–2.98)
fDHA 5	44	4	0	0 (0–8.04)	0	0 (0–8.04)
fDHA 6	76	1	1	1.32 (0.03–7.11)	1	1.32 (0.03–7.11)
Eastern Zone	284	14	0	0 (0–1.29)	1	0.35 (0–1.95)
fDHA 7	71	7	0	0 (0–5.06)	1	1.41 (0.04‐7.60)
fDHA 8	213	7	0	0 (0–1.72)	0	0 (0–1.72)
Central Zone (fDHA 9)	693	21	4	0.58 (0.16–1.47)	2	0.29 (0.03–1.04)
Nova Scotia	1872	93	30	1.60 (1.08–2.28)	25	1.34 (0.87–1.97)

*Note:* Two IgG immunoblots were unreadable due to background staining on the test strip, and were considered ‘undetermined’.

Abbreviations: EIA, enzyme immunoassay; fDHA, former District Health Authority; MTTT, modified two‐tiered testing; WCS, whole cell sonicate.

Using the STTT, 25 of the 30 pepC10/VlsE EIA‐reactive samples were confirmed by the IgG immunoblot. The regional distribution was similar to that of the MTTT analysis, with the highest seroprevalence in the Western Zone, but the seroprevalence was not as high as that determined by the MTTT (Table 1). Overall provincial seropositivity based on STTT was 25 of 1872 tested (1.34%, 95% CI 0.87–1.97). Two immunoblots could not be read due to non‐specific background signal (one each of WCS EIA‐positive and ‐negative), so they were considered undetermined.

## 4 Discussion

Results from this study period demonstrate a higher seropositivity rate to *B. burgdorferi* when compared to results from a prior study performed 10 years ago (from 0.14% to 1.60%) in the Canadian province of Nova Scotia (Hatchette et al. 2015). This increased seropositivity is consistent with an increase in reported cases in Nova Scotia. The Western Zone had high seropositivity, correlating with the highest number of reported cases at 1192 (57.9% of cases in the province) in 2023, for a rate of 582.1 cases per 100,000 population (Nova Scotia Department of Health and Wellness 2024). In fact, when the Western Zone is excluded from the provincial calculation, the seroprevalence is only 0.49% (96% CI 0.18–1.07), as compared to the seropositivity rate of 3.68% in the Western Zone alone. The higher seropositivity of males in this study is consistent with clinical cases reported to public health, where the rate per 100,000 in males was 224.8 compared to 164.7 in females (Nova Scotia Department of Health and Wellness 2024). Although age distribution was important for ensuring sample representativeness, no conclusions could be drawn associating age with seropositivity from our small number of positive tests.

This study has several limitations. The seroprevalence is based on the region where testing was performed and may not reflect where the tick/pathogen exposure occurred. Unfortunately, due to the discontinuation of routine tick surveillance in the province, there is difficulty in ascertaining region‐based prevalence of *I. scapularis* and therefore association of disease with regional vector exposure. In addition, though residual serum has been used in the past to approximate population‐level seroprevalence (Kelly et al. 2002), the potential for bias cannot be eliminated. Residual serum from routine tests could over‐ or under‐estimate exposure to *Ixodes* spp. ticks (and thus the pathogen, via the tick vector) in unanticipated ways. When comparing seroprevalence differences between the current study and that of 2012, it is important to note the change in testing algorithms, from STTT to MTTT and that this may impact the results. Notably, when using the STTT algorithm with the current samples, the increased seroprevalence is approximately ninefold, at 1.34%. These discrepant results likely reflect increased sensitivity of the MTTT assay when compared to STTT, specifically in early LD, where MTTT assays were 25%–28% more sensitive than STTT assays in a Nova Scotian population. Importantly, the MTTT, maintains a high specificity of 99.6% which is essential when interpreting seroprevalence studies (Khan et al. 2022; Davis et al. 2020). Although we sampled residual sera from presumed healthy community‐dwellers, we cannot rule out the possibility that the residual sera were collected from someone with early infection. The specimens were de‐identified at the point of aliquoting, making clinical correlation not possible. Additionally, considering the increased accessibility to prophylactic doxycycline in the community through local pharmacies (Bell et al. 2025), it is possible that we have under‐detected antibodies to *B. burgdorferi* as prophylaxis or early therapy may abrogate an immune response (Hu 2016).

This work demonstrates an increase in seroprevalence to *B. burgdorferi* in Nova Scotia, consistent with the ongoing increase in LD cases seen in the province likely reflecting increases in infected tick populations. Since the first report from a decade ago, three other human pathogens transmitted by *I. scapularis* ( *Anaplasma phagocytophilum* , *Babesia microti*, and Powassan Virus) have been added to the Nova Scotia Notifiable Diseases list, given their increase in clinical cases (Chase and Bonnar 2018; Allehebi et al. 2022) and/or detection in the tick population (Guillot et al. 2022).

Clinicians should continue to consider LD in patients presenting with compatible syndromes and residents and travellers to Nova Scotia should continue to take precautions to protect themselves from *Ixodes* spp. tick exposure, given the increased risk of LD.

## Funding

This work was supported by Research Nova Scotia (2023‐2915).

## Conflicts of Interest

The authors declare no conflicts of interest.

## Data Availability

The data that support the findings of this study are available from the corresponding author upon reasonable request.
